# Supplementary material for: Hybrid Models and Biological Model Reduction with PyDSTool
Source: PLoS Comput Biol. 2012 Aug 9;8(8):e1002628. doi: 10.1371/journal.pcbi.1002628 (PMC3415397; doi:10.1371/journal.pcbi.1002628)
Supplement: Text S4 — Complete source code for the PyDSTool package (version 0.88.120504). Includes API documentation and help files linking to web pages. This file is identical to the current public release on Sourceforge.net. (ZIP) [file pcbi.1002628.s004.zip › PyDSTool/html/PyDSTool.Generator.Vode_ODEsystem'-pysrc.html]

xml version="1.0" encoding="ascii"?


PyDSTool.Generator.Vode\_ODEsystem'


| Home | Trees | Indices | Help | PyDSTool |
| --- | --- | --- | --- | --- |

Package PyDSTool ::
Package Generator ::
Module Vode\_ODEsystem'

[hide private]

[frames] | no frames]

# Source Code for Module PyDSTool.Generator.Vode\_ODEsystem'

```
  1  """VODE integrator for ODE systems, imported from a mild modification of
 
  2  the scipy-wrapped VODE Fortran solver.
 
  3  """ 
  4  from __future__ import division 
  5  
 
  6  from allimports import * 
  7  from PyDSTool.Generator import ODEsystem as ODEsystem 
  8  from baseclasses import Generator, theGenSpecHelper, _pollInputs 
  9  from PyDSTool.utils import * 
 10  from PyDSTool.common import * 
 11  from PyDSTool.scipy_ode import ode 
 12  
 
 13  # Other imports
 
 14  from numpy import Inf, NaN, isfinite, sometrue, alltrue, sign, all, any, \
 
 15       array, zeros, less_equal, transpose, concatenate, asarray, linspace 
 16  try: 
 17      from numpy import unique 
 18  except ImportError: 
 19      # older version of numpy
 
 20      from numpy import unique1d as unique 
 21  import math, random 
 22  from copy import copy, deepcopy 
 23  import os, platform, shutil, sys, gc 
 24  
 
 25  # PSYCO FUNCTIONS DON'T WORK AS VODE CALLBACK FUNCTIONS!
 
 26  #try:
 
 27  #    # use psyco JIT byte-compiler optimization, if available
 
 28  #    import psyco
 
 29  #    HAVE_PSYCO = True
 
 30  #except ImportError:
 
 31  #    HAVE_PSYCO = False
 
 32  HAVE_PSYCO = False 
 33  
 
 34  
 


35 -class Vode_ODEsystem(ODEsystem):


36      """Wrapper for VODE, from SciPy.
 
 37  
 
 38      Uses Python target language only for functional specifications.""" 
 39  
 


40 -    def __init__(self, kw):


41          ODEsystem.__init__(self, kw) 
 42          self._paraminfo = {'init_step': 'Fixed step size for time mesh.',
 
 43                             'strictdt': 'Boolean determining whether to evenly space time mesh (default=False), or to use exactly dt spacing.',
 
 44                             'stiff': 'Boolean to activate the BDF method, otherwise Adams method used. Default False.',
 
 45                             'use_special': "Switch for using special times",
 
 46                             'specialtimes': "List of special times to use during integration",} 
 47          self.diagnostics._errorcodes = \
 
 48                  {0: 'Unrecognized error code returned (see stderr output)',
 
 49                  -1: 'Excess work done on this call. (Perhaps wrong method MF.)',
 
 50                  -2: 'Excess accuracy requested. (Tolerances too small.)',
 
 51                  -3: 'Illegal input detected. (See printed message.)',
 
 52                  -4: 'Repeated error test failures. (Check all input.)',
 
 53                  -5: 'Repeated convergence failures. (Perhaps bad'
 
 54                      ' Jacobian supplied or wrong choice of method MF or tolerances.)',
 
 55                  -6: 'Error weight became zero during problem. (Solution'
 
 56                      ' component i vanished, and ATOL or ATOL(i) = 0.)'
 
 57                  } 
 58          self.diagnostics.outputStatsInfo = {'errorStatus': 'Error status on completion.'} 
 59          # note: VODE only supports array atol, not rtol.
 
 60          algparams_def = {'poly_interp': False,
 
 61                           'rtol': 1e-9,
 
 62                           'atol': [1e-12 for dimix in xrange(self.dimension)],
 
 63                           'stiff': False,
 
 64                           'max_step': 0.0,
 
 65                           'min_step': 0.0,
 
 66                           'init_step': 0.01,
 
 67                           'max_pts': 1000000,
 
 68                           'strictdt': False,
 
 69                           'use_special': False,
 
 70                           'specialtimes': []
 
 71                           } 
 72          for k, v in algparams_def.iteritems(): 
 73              if k not in self.algparams: 
 74                  self.algparams[k] = v

 75  
 
 76  
 


77 -    def addMethods(self):


78          # override to add _solver function
 
 79          ODEsystem.addMethods(self, usePsyco=False) 
 80          if self.haveJacobian(): 
 81              self._solver = ode(getattr(self,self.funcspec.spec[1]),
 
 82                              getattr(self,self.funcspec.auxfns["Jacobian"][1])) 
 83              self._funcreg['_solver'] = ('self',
 
 84                   'ode(getattr(self,self.funcspec.spec[1]),' \
 
 85                     + 'getattr(self,self.funcspec.auxfns["Jacobian"][1]))') 
 86          else: 
 87              self._solver = ode(getattr(self,self.funcspec.spec[1])) 
 88              self._funcreg['_solver'] = ('self', 'ode(getattr(self,' \
 
 89                                                    + 'self.funcspec.spec[1]))')

 90  
 
 91  
 


92 -    def _debug_snapshot(self, solver, dt, inputlist):


93          ivals = [i(solver.t) for i in inputlist] 
 94          s = "\n***************\nNew t, x, inputs: " + " ".join([str(s) for s in (solver.t,solver.y,ivals)]) 
 95          s += "\ndt="+str(dt)+" f_params="+str(solver.f_params)+" dx/dt=" 
 96          s += str(solver.f(solver.t, solver.y, sortedDictValues(self.pars)+ivals)) 
 97          if solver.t > 7: 
 98              s += "\nfirst, max, min steps =" + str([solver.first_step, solver.max_step, solver.min_step]) 
 99          return s

100  
 


101 -    def compute(self, trajname, dirn='f', ics=None):


102          continue_integ = ODEsystem.prepDirection(self, dirn) 
103          if self._dircode == -1: 
104              raise NotImplementedError('Backwards integration is not implemented') 
105          if ics is not None: 
106              self.set(ics=ics) 
107          self.validateICs() 
108          self.diagnostics.clearWarnings() 
109          self.diagnostics.clearErrors() 
110          pnames = sortedDictKeys(self.pars) 
111          xnames = self._var_ixmap  # ensures correct order 
112          # Check i.c.'s are well defined (finite)
 
113          self.checkInitialConditions() 
114          if self.algparams['stiff']: 
115              methstr = 'bdf' 
116              methcode = 2 
117          else: 
118              methstr = 'adams' 
119              methcode = 1 
120          haveJac = int(self.haveJacobian()) 
121          if isinstance(self.algparams['atol'], list): 
122              if len(self.algparams['atol']) != self.dimension: 
123                  raise ValueError('atol list must have same length as phase '
 
124                                   'dimension') 
125          else: 
126              atol = self.algparams['atol'] 
127              self.algparams['atol'] = [atol for dimix in xrange(self.dimension)] 
128          indepdom0, indepdom1 = self.indepvariable.depdomain.get() 
129          if continue_integ: 
130              if indepdom0 > self._solver.t: 
131                  print "Previous end time is %f"%self._solver.t 
132                  raise ValueError("Start time not correctly updated for "
 
133                                   "continuing orbit") 
134              x0 = self._solver.y 
135              indepdom0 = self._solver.t 
136              self.indepvariable.depdomain.set((indepdom0,indepdom1)) 
137          else: 
138              x0 = sortedDictValues(self.initialconditions,
 
139                                          self.funcspec.vars) 
140          t0 = indepdom0 
141          if self.algparams['use_special']: 
142              tmesh_special = list(self.algparams['specialtimes']) 
143              if continue_integ: 
144                  if self._solver.t not in tmesh_special: 
145                      raise ValueError("Invalid time to continue integration:"
 
146                                       "it is not in 'special times'") 
147                  tmesh_special = tmesh_special[tmesh_special.index(self._solver.t):] 
148              try: 
149                  dt = min([tmesh_special[1]-t0, self.algparams['init_step']]) 
150              except: 
151                  raise ValueError("Invalid list of special times") 
152              if not isincreasing(tmesh_special): 
153                  raise ValueError("special times must be given in increasing "
 
154                                   "order") 
155              if self.indepvariable.depdomain.contains(t0) is notcontained or \
 
156                self.indepvariable.depdomain.contains(tmesh_special[-1]) is notcontained: 
157                  raise PyDSTool_BoundsError("special times were outside of independent "
 
158                                   "variable bounds") 
159          else: 
160              tmesh_special = [] 
161              dt = self.algparams['init_step'] 
162          # speed up repeated access to solver by making a temp name for it
 
163          solver = self._solver 
164          if solver._integrator is None: 
165              # Banded Jacobians not yet supported
 
166              #
 
167              # start a new integrator, because method may have been
 
168              # switched
 
169              solver.set_integrator('vode', method=methstr,
 
170                                   rtol=self.algparams['rtol'],
 
171                                   atol=self.algparams['atol'],
 
172                                   nsteps=self.algparams['max_pts'],
 
173                                   max_step=self.algparams['max_step'],
 
174                                   min_step=self.algparams['min_step'],
 
175                                   first_step=dt/2.,
 
176                                   with_jacobian=haveJac) 
177          else: 
178              solver.with_jacobian = haveJac 
179  #            self.mu = lband
 
180  #            self.ml = uband
 
181              solver.rtol = self.algparams['rtol'] 
182              solver.atol = self.algparams['atol'] 
183              solver.method = methcode 
184  #            self.order = order
 
185              solver.nsteps = self.algparams['max_pts'] 
186              solver.max_step = self.algparams['max_step'] 
187              solver.min_step = self.algparams['min_step'] 
188              solver.first_step = dt/2. 
189          solver.set_initial_value(x0, t0) 
190          ## Broken code for going backwards (doesn't respect 'continue' option
 
191          ##  either)
 
192  ##            if self._dircode == 1:
 
193  ##                solver.set_initial_value(x0, t0)
 
194  ##            else:
 
195  ##                solver.set_initial_value(x0, indepdom1)
 
196          # wrap up each dictionary initial value as a singleton list
 
197          alltData = [t0] 
198          allxDataDict = dict(zip(xnames, map(listid, x0))) 
199          plist = sortedDictValues(self.pars) 
200          extralist = copy(plist) 
201          if self.inputs: 
202              # inputVarList is a list of Variables
 
203              inames = sortedDictKeys(self.inputs) 
204              listend = self.numpars + len(self.inputs) 
205              inputVarList = sortedDictValues(self.inputs) 
206              ilist = _pollInputs(inputVarList, alltData[0]+self.globalt0,
 
207                                 self.checklevel) 
208          else: 
209              ilist = [] 
210              inames = [] 
211              listend = self.numpars 
212              inputVarList = [] 
213          extralist.extend(ilist) 
214          solver.set_f_params(extralist) 
215          if haveJac: 
216              solver.set_jac_params(extralist) 
217          do_poly = self.algparams['poly_interp'] 
218          if do_poly: 
219              rhsfn = getattr(self, self.funcspec.spec[1]) 
220              dx0 = rhsfn(t0, x0, extralist) 
221              alldxDataDict = dict(zip(xnames, map(listid, dx0))) 
222          auxvarsfn = getattr(self,self.funcspec.auxspec[1]) 
223          strict = self.algparams['strictdt'] 
224          # Make t mesh if it wasn't given as 'specialtimes'
 
225          if not all(isfinite(self.indepvariable.depdomain.get())): 
226              print "Time domain was: ", self.indepvariable.depdomain.get() 
227              raise ValueError("Ensure time domain is finite") 
228          if dt == indepdom1 - indepdom0: 
229              # single-step integration required
 
230              # special times will not have been set (unless trivially
 
231              # they are [indepdom0, indepdom1])
 
232              tmesh = [indepdom0, indepdom1] 
233          else: 
234              notDone = True 
235              repeatTol = 10 
236              count = 0 
237              while notDone and count <= repeatTol: 
238                  try: 
239                      tmesh = self.indepvariable.depdomain.sample(dt,
 
240                                            strict=strict,
 
241                                            avoidendpoints=self.checklevel>2) 
242                      notDone = False 
243                  except AssertionError: 
244                      count += 1 
245                      dt = dt/3.0 
246              if count == repeatTol: 
247                  raise AssertionError("Supplied time step is too large for "
 
248                                       "selected time interval") 
249              # incorporate tmesh_special, if used, ensuring uniqueness
 
250              if tmesh_special != []: 
251                  tmesh.extend(tmesh_special) 
252                  tmesh = list(unique(tmesh)) 
253                  tmesh.sort() 
254              if len(tmesh)<=2: 
255                  # safety net, in case too few points in mesh
 
256                  # too few points unless we can add endpoint
 
257                  if tmesh[-1] != indepdom1: 
258                      # dt too large for tmesh to have more than one point
 
259                      tmesh.append(indepdom1) 
260              if not strict:  # get actual time step used 
261                  # don't use [0] in case avoided end points
 
262                  try: 
263                      dt = tmesh[2]-tmesh[1] 
264                  except IndexError: 
265                      # can't avoid end points for such a small mesh
 
266                      dt = tmesh[1]-tmesh[0] 
267          #if self.eventstruct.query(['lowlevel']) != []:
 
268          #    raise ValueError("Only high level events can be passed to VODE")
 
269          eventslist = self.eventstruct.query(['active', 'notvarlinked']) 
270          termevents = self.eventstruct.query(['term'], eventslist) 
271          # reverse time by reversing mesh doesn't work
 
272  ##        if self._dircode == -1:
 
273  ##            tmesh.reverse()
 
274          tmesh.pop(0)  # get rid of first entry for initial condition 
275          xnames = self.funcspec.vars 
276          # storage of all auxiliary variable data
 
277          allaDataDict = {} 
278          anames = self.funcspec.auxvars 
279          avals = auxvarsfn(t0, x0, extralist) 
280          for aix in range(len(anames)): 
281              aname = anames[aix] 
282              try: 
283                  allaDataDict[aname] = [avals[aix]] 
284              except IndexError: 
285                  print "\nVODE generator: There was a problem evaluating " \
 
286                        + "an auxiliary variable" 
287                  print "Debug info: avals (length", len(avals), ") was ", avals 
288                  print "Index out of range was ", aix 
289                  print self.funcspec.auxspec[1] 
290                  print hasattr(self, self.funcspec.auxspec[1]) 
291                  print "Args were:", [t0, x0, extralist] 
292                  raise 
293          # Initialize signs of event detection objects at IC
 
294          self.setEventICs(self.initialconditions, self.globalt0) 
295          if self.inputs: 
296              parsinps = copy(self.pars) 
297              parsinps.update(dict(zip(inames,ilist))) 
298          else: 
299              parsinps = self.pars 
300          if eventslist != []: 
301              dataDict = copy(self.initialconditions) 
302              dataDict.update(dict(zip(anames, avals))) 
303              dataDict['t'] = t0 
304              evsflagged = self.eventstruct.pollHighLevelEvents(None,
 
305                                                              dataDict,
 
306                                                              parsinps,
 
307                                                              eventslist) 
308              if len(evsflagged) > 0: 
309                  raise RuntimeError("Some events flagged at initial condition") 
310              if continue_integ: 
311                  # revert to prevprevsign, since prevsign changed after call
 
312                  self.eventstruct.resetHighLevelEvents(t0, eventslist, 'prev') 
313              elif self._for_hybrid_DS: 
314                  # self._for_hybrid_DS is set internally by HybridModel class
 
315                  # to ensure not to reset events, because they may be about to
 
316                  # flag on first step if previous hybrid state was the same
 
317                  # generator and, for example, two variables are synchronizing
 
318                  # so that their events get very close together.
 
319                  # Just reset the starttimes of these events
 
320                  for evname, ev in eventslist: 
321                      ev.starttime = t0 
322              else: 
323                  # default state is a one-off call to this generator
 
324                  self.eventstruct.resetHighLevelEvents(t0, eventslist, None) 
325                  self.eventstruct.validateEvents(self.funcspec.vars + \
 
326                                              self.funcspec.auxvars + \
 
327                                              self.funcspec.inputs + \
 
328                                              ['t'], eventslist) 
329          # temp storage of first time at which terminal events found
 
330          # (this is used for keeping the correct end point of new mesh)
 
331          first_found_t = None 
332          # list of precise non-terminal events to be resolved after integration
 
333          nontermprecevs = [] 
334          evnames = [ev[0] for ev in eventslist] 
335          lastevtime = {}.fromkeys(evnames, None) 
336          # initialize new event info dictionaries
 
337          Evtimes = {} 
338          Evpoints = {} 
339          if continue_integ: 
340              for evname in evnames: 
341                  try: 
342                      # these are in global time, so convert to local time
 
343                      lastevtime[evname] = self.eventstruct.Evtimes[evname][-1] \
 
344                                             - self.globalt0 
345                  except (IndexError, KeyError): 
346                      # IndexError: Evtimes[evname] was None
 
347                      # KeyError: Evtimes does not have key evname
 
348                      pass 
349          for evname in evnames: 
350              Evtimes[evname] = [] 
351              Evpoints[evname] = [] 
352          # temp storage for repeatedly used object attributes (for lookup efficiency)
 
353          depdomains = dict(zip(range(self.dimension),
 
354                          [self.variables[xn].depdomain for xn in xnames])) 
355          # Main integration loop
 
356          num_points = 0 
357          breakwhile = False 
358          while not breakwhile: 
359              try: 
360                  new_t = tmesh.pop(0)  # this destroys tmesh for future use 
361              except IndexError: 
362                  # empty
 
363                  break 
364              try: 
365                  errcode = solver.integrate(new_t) 
366              except: 
367                  print "Error calling right hand side function:" 
368                  self.showSpec() 
369                  print "Numerical traceback information (current state, " \
 
370                        + "parameters, etc.)" 
371                  print "in generator dictionary 'traceback'" 
372                  self.traceback = {'vars': dict(zip(xnames,solver.y)),
 
373                                    'pars': dict(zip(pnames,plist)),
 
374                                    'inputs': dict(zip(inames,ilist)),
 
375                                    self.indepvariable.name: new_t} 
376                  raise 
377              avals = auxvarsfn(new_t, solver.y, extralist) 
378              # Uncomment the following assertion for debugging
 
379  #            assert all([isfinite(a) for a in avals]), \
 
380  #               "Some auxiliary variable values not finite"
 
381              if eventslist != []: 
382                  dataDict = dict(zip(xnames,solver.y)) 
383                  dataDict.update(dict(zip(anames,avals))) 
384                  dataDict['t'] = new_t 
385                  if self.inputs: 
386                      parsinps = copy(self.pars) 
387                      parsinps.update(dict(zip(inames,
 
388                                _pollInputs(inputVarList, new_t+self.globalt0,
 
389                                            self.checklevel)))) 
390                  else: 
391                      parsinps = self.pars 
392                  evsflagged = self.eventstruct.pollHighLevelEvents(None,
 
393                                                              dataDict,
 
394                                                              parsinps,
 
395                                                              eventslist) 
396  ##                print new_t, evsflagged
 
397  ##                evsflagged = [ev for ev in evsflagged if solver.t-indepdom0 > ev[1].eventinterval]
 
398                  termevsflagged = filter(lambda e: e in evsflagged, termevents) 
399                  nontermevsflagged = filter(lambda e: e not in termevsflagged,
 
400                                             evsflagged) 
401                  # register any non-terminating events in the warnings
 
402                  # list, unless they are 'precise' in which case flag
 
403                  # them to be resolved after integration completes
 
404                  if len(nontermevsflagged) > 0: 
405                      evnames = [ev[0] for ev in nontermevsflagged] 
406                      precEvts = self.eventstruct.query(['precise'],
 
407                                                            nontermevsflagged) 
408                      prec_evnames = [e[0] for e in precEvts] 
409                      # first register non-precise events
 
410                      nonprecEvts = self.eventstruct.query(['notprecise'],
 
411                                                           nontermevsflagged) 
412                      nonprec_evnames = [e[0] for e in nonprecEvts] 
413                      # only record events if they have not been previously
 
414                      # flagged within their event interval
 
415                      if nonprec_evnames != []: 
416                          temp_names = [] 
417                          for evname, ev in nonprecEvts: 
418                              prevevt_time = lastevtime[evname] 
419                              if prevevt_time is None: 
420                                  ignore_ev = False 
421                              else: 
422                                  if solver.t-prevevt_time < ev.eventinterval: 
423                                      ignore_ev = True 
424                                  else: 
425                                      ignore_ev = False 
426                              if not ignore_ev: 
427                                  temp_names.append(evname) 
428                                  lastevtime[evname] = solver.t 
429                          self.diagnostics.warnings.append((W_NONTERMEVENT,
 
430                                       (solver.t, temp_names))) 
431                          for evname in temp_names: 
432                              Evtimes[evname].append(solver.t) 
433                              xv = solver.y 
434                              av = array(avals) 
435                              Evpoints[evname].append(concatenate((xv, av))) 
436                      for evname, ev in precEvts: 
437                          # only record events if they have not been previously
 
438                          # flagged within their event interval
 
439                          prevevt_time = lastevtime[evname] 
440                          if prevevt_time is None: 
441                              ignore_ev = False 
442                          else: 
443                              if solver.t-dt-prevevt_time < ev.eventinterval: 
444                                  ignore_ev = True 
445                              else: 
446                                  ignore_ev = False 
447                          if not ignore_ev: 
448                              nontermprecevs.append((solver.t-dt, solver.t, (evname, ev))) 
449                              # be conservative as to where the event is, so
 
450                              # that don't miss any events.
 
451                              lastevtime[evname] = solver.t-dt 
452                          ev.reset() #ev.prevsign = None # 
453                  do_termevs = [] 
454                  if termevsflagged != []: 
455                      # only record events if they have not been previously
 
456                      # flagged within their event interval
 
457                      for e in termevsflagged: 
458                          prevevt_time = lastevtime[e[0]] 
459  ##                        print "Event %s flagged."%e[0]
 
460  ##                        print "  ... last time was ", prevevt_time
 
461  ##                        print "  ... event interval = ", e[1].eventinterval
 
462  ##                        print "  ... t = %f, dt = %f"%(solver.t, dt)
 
463                          if prevevt_time is None: 
464                              ignore_ev = False 
465                          else: 
466  ##                            print "  ... comparison = %f < %f"%(solver.t-dt-prevevt_time, e[1].eventinterval)
 
467                              if solver.t-dt-prevevt_time < e[1].eventinterval: 
468                                  ignore_ev = True 
469  ##                                print "VODE ignore ev"
 
470                              else: 
471                                  ignore_ev = False 
472                          if not ignore_ev: 
473                              do_termevs.append(e) 
474                  if len(do_termevs) > 0: 
475                      # >= 1 active terminal event flagged at this time point
 
476                      if all([not ev[1].preciseFlag for ev in do_termevs]): 
477                          # then none of the events specify greater accuracy
 
478                          # register the event in the warnings
 
479                          evnames = [ev[0] for ev in do_termevs] 
480                          self.diagnostics.warnings.append((W_TERMEVENT, \
 
481                                               (solver.t, evnames))) 
482                          for evname in evnames: 
483                              Evtimes[evname].append(solver.t) 
484                              xv = solver.y 
485                              av = array(avals) 
486                              Evpoints[evname].append(concatenate((xv, av))) 
487                          # break while loop after appending t, x
 
488                          breakwhile = True 
489                      else: 
490                          # find which are the 'precise' events that flagged
 
491                          precEvts = self.eventstruct.query(['precise'],
 
492                                                            do_termevs) 
493                          # these events have flagged once so eventdelay has
 
494                          # been used. now switch it off while finding event
 
495                          # precisely (should be redundant after change to
 
496                          # eventinterval and eventdelay parameters)
 
497                          evnames = [ev[0] for ev in precEvts] 
498                          if first_found_t is None: 
499  ##                            print "first time round at", solver.t
 
500                              numtries = 0 
501                              first_found_t = solver.t 
502                              restore_evts = deepcopy(precEvts) 
503                              minbisectlimit = min([ev[1].bisectlimit for ev in precEvts]) 
504                              for ev in precEvts: 
505                                  ev[1].eventdelay = 0. 
506                          else: 
507                              numtries += 1 
508  ##                            print "time round: ", numtries
 
509                              if numtries > minbisectlimit: 
510                                  self.diagnostics.warnings.append((W_BISECTLIMIT,
 
511                                                        (solver.t, evnames))) 
512                                  breakwhile = True 
513  
 
514                          # get previous time point
 
515                          if len(alltData)>=1: 
516                              # take one step back -> told, which will
 
517                              # get dt added back to first new meshpoint
 
518                              # (solver.t is the step *after* the event was
 
519                              # detected)
 
520                              told = solver.t-dt 
521                          else: 
522                              raise ValueError("Event %s found too "%evnames[0]+\
 
523                                   "close to local start time: try decreasing "
 
524                                   "initial step size (current size is "
 
525                                   "%f @ t=%f)"%(dt,solver.t+self.globalt0)) 
526  
 
527                          # absolute tolerance check on event function values (not t)
 
528                          in_tols = [abs(e[1].fval) < e[1].eventtol for e in precEvts] 
529                          if all(in_tols): 
530  ##                            print "Registering event:", dt_min, dt
 
531                              # register the event in the warnings
 
532                              self.diagnostics.warnings.append((W_TERMEVENT,
 
533                                               (solver.t, evnames))) 
534                              for evname in evnames: 
535                                  Evtimes[evname].append(solver.t) 
536                                  xv = solver.y 
537                                  av = array(avals) 
538                                  Evpoints[evname].append(concatenate((xv, av))) 
539                              # Cannot continue -- dt_min no smaller than
 
540                              # previous dt. If this is more than the first time
 
541                              # in this code then have found the event to within
 
542                              # the minimum 'precise' event's eventtol, o/w need
 
543                              # to set eventtol smaller.
 
544                              # Before exiting event-finding loop, reset prevsign
 
545                              # of flagged events
 
546                              self.eventstruct.resetHighLevelEvents(0,
 
547                                                               precEvts) 
548                              breakwhile = True  # while loop, but append point first 
549                          if not breakwhile: 
550                              dt_new = dt/5.0 
551                              # calc new tmesh
 
552                              trangewidth = 2*dt #first_found_t - told 
553                              numpoints = int(math.ceil(trangewidth/dt_new)) 
554                              # choose slightly smaller dt to fit trange exactly
 
555                              dt = trangewidth/numpoints 
556  
 
557                              tmesh = [told + i*dt for i in xrange(1, numpoints+1)] 
558                              # linspace version is *much* slower for numpoints ~ 10 and 100
 
559                              #tmesh = list(told+linspace(dt, numpoints*dt, numpoints))
 
560  
 
561                              # reset events according to new time mesh,
 
562                              # setting known previous event state to be their
 
563                              # "no event found" state
 
564                              self.eventstruct.resetHighLevelEvents(told,
 
565                                                                    precEvts,
 
566                                                                    state='off') 
567                              # build new ic with last good values (at t=told)
 
568                              if len(alltData)>1: 
569                                  new_ic = [allxDataDict[xname][-1] \
 
570                                              for xname in xnames] 
571                              else: 
572                                  new_ic = x0 
573                              # reset integrator
 
574                              solver.set_initial_value(new_ic, told) 
575                              extralist[self.numpars:listend] = _pollInputs(inputVarList,
 
576                                                      told+self.globalt0, self.checklevel) 
577                              solver.set_f_params(extralist) 
578                              # continue integrating over new mesh
 
579                              continue  # while 
580              # after events have had a chance to be detected at a state boundary
 
581              # we check for any that have not been caught by an event (will be
 
582              # much less accurately determined)
 
583              if not breakwhile: 
584                  # only here if a terminal event hasn't just flagged
 
585                  for xi in xrange(self.dimension): 
586                      if not self.contains(depdomains[xi],
 
587                                       solver.y[xi],
 
588                                       self.checklevel): 
589                          self.diagnostics.warnings.append((W_TERMSTATEBD,
 
590                                      (solver.t,
 
591                                       xnames[xi],solver.y[xi],
 
592                                       depdomains[xi].get()))) 
593                          breakwhile = True 
594                          break  # for loop 
595                  if breakwhile: 
596                      break  # while loop 
597              alltData.append(solver.t) 
598              if do_poly: 
599                  dxvals = rhsfn(solver.t, solver.y, extralist) 
600                  for xi, xname in enumerate(xnames): 
601                      allxDataDict[xname].append(solver.y[xi]) 
602                      alldxDataDict[xname].append(dxvals[xi]) 
603              else: 
604                  for xi, xname in enumerate(xnames): 
605                      allxDataDict[xname].append(solver.y[xi]) 
606              for aix, aname in enumerate(anames): 
607                  allaDataDict[aname].append(avals[aix]) 
608              num_points += 1 
609              if not breakwhile: 
610                  try: 
611                      extralist[self.numpars:listend] = [f(solver.t+self.globalt0,
 
612                                                           self.checklevel) \
 
613                                                    for f in inputVarList] 
614                  except ValueError: 
615                      print 'External input call caused value out of range error:',\
 
616                            't = ', solver.t 
617                      for f in inputVarList: 
618                          if f.diagnostics.hasWarnings(): 
619                              print 'External input variable %s out of range:'%f.name 
620                              print '   t = ', repr(f.diagnostics.warnings[-1][0]), ', ', \
 
621                                    f.name, ' = ', repr(f.diagnostics.warnings[-1][1]) 
622                      raise 
623                  except AssertionError: 
624                      print 'External input call caused t out of range error: t = ', \
 
625                            solver.t 
626                      raise 
627                  solver.set_f_params(extralist) 
628                  breakwhile = not solver.successful() 
629          # Check that any terminal events found terminated the code correctly
 
630          if first_found_t is not None: 
631              # ... then terminal events were found. Those that were 'precise' had
 
632              # their 'eventdelay' attribute temporarily set to 0. It now should
 
633              # be restored.
 
634              for evname1, ev1 in termevents: 
635                  # restore_evts are copies of the originally flagged 'precise'
 
636                  # events
 
637                  for evname2, ev2 in restore_evts: 
638                      if evname2 == evname1: 
639                          ev1.eventdelay = ev2.eventdelay 
640              try: 
641                  if self.diagnostics.warnings[-1][0] not in [W_TERMEVENT,
 
642                                                              W_TERMSTATEBD]: 
643                      print "t =", solver.t 
644                      print "state =", dict(zip(xnames,solver.y)) 
645                      raise RuntimeError("Event finding code for terminal event "
 
646                                         "failed in Generator " + self.name + \
 
647                                         ": try decreasing eventdelay or "
 
648                                         "eventinterval below eventtol, or the "
 
649                                         "atol and rtol parameters") 
650              except IndexError: 
651                  info(self.diagnostics.outputStats, "Output statistics") 
652                  print "t =", solver.t 
653                  print "x =", solver.y 
654                  raise RuntimeError("Event finding failed in Generator " + \
 
655                                     self.name + ": try decreasing eventdelay "
 
656                                     "or eventinterval below eventtol, or the "
 
657                                     "atol and rtol parameters") 
658          # Package up computed trajectory in Variable variables
 
659          # Add external inputs warnings to self.diagnostics.warnings, if any
 
660          for f in inputVarList: 
661              for winfo in f.diagnostics.warnings: 
662                  self.diagnostics.warnings.append((W_NONTERMSTATEBD,
 
663                                       (winfo[0], f.name, winfo[1],
 
664                                        f.depdomain.get()))) 
665          # check for non-unique terminal event
 
666          termcount = 0 
667          for (w,i) in self.diagnostics.warnings: 
668              if w == W_TERMEVENT or w == W_TERMSTATEBD: 
669                  termcount += 1 
670                  if termcount > 1: 
671                      self.diagnostics.errors.append((E_NONUNIQUETERM,
 
672                                                      (alltData[-1], i[1]))) 
673          # uncomment the following lines for debugging
 
674  #        assert len(alltData) == len(allxDataDict.values()[0]) \
 
675  #             == len(allaDataDict.values()[0]), "Output data size mismatch"
 
676  #        for val_list in allaDataDict.values():
 
677  #            assert all([isfinite(x) for x in val_list])
 
678          # Create variables (self.variables contains no actual data)
 
679          # These versions of the variables are only final if no non-terminal
 
680          # events need to be inserted.
 
681          variables = copyVarDict(self.variables) 
682          for x in xnames: 
683              if len(alltData) > 1: 
684                  if do_poly: 
685                      xvals = array([allxDataDict[x], alldxDataDict[x]]).T 
686                      interp = PiecewisePolynomial(alltData, xvals, 2) 
687                  else: 
688                      xvals = allxDataDict[x] 
689                      interp = interp1d(alltData, xvals) 
690                  variables[x] = Variable(interp, 't', x, x) 
691              else: 
692                  print "Error in Generator:", self.name 
693                  print "t = ", alltData 
694                  print "x = ", allxDataDict 
695                  raise PyDSTool_ValueError("Fewer than 2 data points computed") 
696          for a in anames: 
697              if len(alltData) > 1: 
698                  variables[a] = Variable(interp1d(alltData, allaDataDict[a]),
 
699                                          't', a, a) 
700              else: 
701                  print "Error in Generator:", self.name 
702                  print "t = ", alltData 
703                  print "x = ", allxDataDict 
704                  raise PyDSTool_ValueError("Fewer than 2 data points computed") 
705          # Resolve non-terminal 'precise' events that were flagged, using the
 
706          # variables created. Then, add them to a new version of the variables.
 
707          ntpe_tdict = {} 
708          for (et0,et1,e) in nontermprecevs: 
709              lost_evt = False 
710              # problem if eventinterval > et1-et0 !!
 
711              # was: search_dt = max((et1-et0)/5,e[1].eventinterval)
 
712              search_dt = (et1-et0)/5 
713              try: 
714                  et_precise_list = e[1].searchForEvents(trange=[et0,et1],
 
715                                                dt=search_dt,
 
716                                                checklevel=self.checklevel,
 
717                                                parDict=self.pars,
 
718                                                vars=variables,
 
719                                                inputs=self.inputs,
 
720                                                abseps=self._abseps,
 
721                                                eventdelay=False,
 
722                                                globalt0=self.globalt0) 
723              except (ValueError, PyDSTool_BoundsError): 
724                  # dt too large for trange, e.g. if event tol is smaller than time mesh
 
725                  et_precise_list = [(et0, (et0, et1))] 
726              if et_precise_list == []: 
727                  lost_evt = True 
728              for et_precise in et_precise_list: 
729                  if et_precise[0] is not None: 
730                      if et_precise[0] in ntpe_tdict: 
731                          # add event name at this time (that already exists in the dict)
 
732                          ntpe_tdict[et_precise[0]].append(e[0]) 
733                      else: 
734                          # add event name at this time (when time is not already in dict)
 
735                          ntpe_tdict[et_precise[0]] = [e[0]] 
736                  else: 
737                      lost_evt = True 
738              if lost_evt: 
739                  print "Error: A non-terminal, 'precise' event was lost -- did you reset", 
740                  print "events prior to integration?" 
741                  raise PyDSTool_ExistError("Internal error: A non-terminal, "
 
742                      "'precise' event '%s' was lost after integration!"%e[0]) 
743          # add non-terminal event points to variables
 
744          if ntpe_tdict != {}: 
745              # find indices of times at which event times will be inserted
 
746              tix = 0 
747              evts = ntpe_tdict.keys() 
748              evts.sort() 
749              for evix in xrange(len(evts)): 
750                  evt = evts[evix] 
751                  evnames = ntpe_tdict[evt] 
752                  self.diagnostics.warnings.append((W_NONTERMEVENT, (evt, evnames))) 
753                  xval = [variables[x](evt) for x in xnames] 
754                  ilist = _pollInputs(inputVarList, evt+self.globalt0,
 
755                                 self.checklevel) 
756                  # find accurate dx and aux vars value at this time
 
757                  if do_poly: 
758                      dxval = rhsfn(evt, xval, plist+ilist) 
759                  aval = list(auxvarsfn(evt, xval, plist+ilist)) 
760                  for evname in evnames: 
761                      Evtimes[evname].append(evt) 
762                      Evpoints[evname].append(array(xval+aval)) 
763                  tcond = less_equal(alltData[tix:], evt).tolist() 
764                  try: 
765                      tix = tcond.index(0) + tix  # lowest index for t > evt 
766                      do_insert = (alltData[tix-1] != evt) 
767                  except ValueError: 
768                      # evt = last t value so no need to add it
 
769                      do_insert = False 
770                  if do_insert: 
771                      alltData.insert(tix, evt) 
772                      for ai, a in enumerate(anames): 
773                          allaDataDict[a].insert(tix, aval[ai]) 
774                      if do_poly: 
775                          for xi, x in enumerate(xnames): 
776                              allxDataDict[x].insert(tix, xval[xi]) 
777                              alldxDataDict[x].insert(tix, dxval[xi]) 
778                      else: 
779                          for xi, x in enumerate(xnames): 
780                              allxDataDict[x].insert(tix, xval[xi]) 
781              for x in xnames: 
782                  if do_poly: 
783                      # use asarray in case points added to sequences as a list
 
784                      xvals = array([asarray(allxDataDict[x]),
 
785                                     asarray(alldxDataDict[x])]).T 
786                      interp = PiecewisePolynomial(alltData, xvals, 2) 
787                  else: 
788                      xvals = allxDataDict[x] 
789                      interp = interp1d(alltData, xvals) 
790                  variables[x] = Variable(interp, 't', x, x) 
791              for a in anames: 
792                  variables[a] = Variable(interp1d(alltData, allaDataDict[a]),
 
793                                              't', a, a) 
794          self.diagnostics.outputStats = {'last_step': dt,
 
795                              'num_fcns': num_points,
 
796                              'num_steps': num_points,
 
797                              'errorStatus': errcode
 
798                              } 
799          if solver.successful(): 
800              #self.validateSpec()
 
801              for evname, evtlist in Evtimes.iteritems(): 
802                  try: 
803                      self.eventstruct.Evtimes[evname].extend([et+self.globalt0 \
 
804                                              for et in evtlist]) 
805                  except KeyError: 
806                      self.eventstruct.Evtimes[evname] = [et+self.globalt0 \
 
807                                              for et in evtlist] 
808              # build event pointset information (reset previous trajectory's)
 
809              self.trajevents = {} 
810              for (evname, ev) in eventslist: 
811                  evpt = Evpoints[evname] 
812                  if evpt == []: 
813                      self.trajevents[evname] = None 
814                  else: 
815                      evpt = transpose(array(evpt)) 
816                      self.trajevents[evname] = Pointset({'coordnames': xnames+anames,
 
817                                                 'indepvarname': 't',
 
818                                                 'coordarray': evpt,
 
819                                                 'indepvararray': Evtimes[evname],
 
820                                                 'indepvartype': float}) 
821              self.defined = True 
822              return Trajectory(trajname, variables.values(),
 
823                                abseps=self._abseps, globalt0=self.globalt0,
 
824                                checklevel=self.checklevel,
 
825                                FScompatibleNames=self._FScompatibleNames,
 
826                                FScompatibleNamesInv=self._FScompatibleNamesInv,
 
827                                events=self.trajevents,
 
828                                modelNames=self.name,
 
829                                modelEventStructs=self.eventstruct) 
830          else: 
831              try: 
832                  self.diagnostics.errors.append((E_COMPUTFAIL, (solver.t,
 
833                                      self.diagnostics._errorcodes[errcode]))) 
834              except TypeError: 
835                  # e.g. when errcode has been used to return info list
 
836                  print "Error information: ", errcode 
837                  self.diagnostics.errors.append((E_COMPUTFAIL, (solver.t,
 
838                                      self.diagnostics._errorcodes[0]))) 
839              self.defined = False

840  
 
841  
 


842 -    def Rhs(self, t, xdict, pdict=None, asarray=True):


843          """asarray is an unused, dummy argument for compatibility with Model.Rhs""" 
844          # don't need to convert names to FS-compatible as they sort
 
845          # the same
 
846          # also, ensure xdict doesn't contain elements like array([4.1]) instead of 4
 
847          x = [float(val) for val in sortedDictValues(filteredDict(xdict,
 
848                                                                   self.funcspec.vars))] 
849          if pdict is None: 
850              pdict = self.pars 
851          p = sortedDictValues(pdict) 
852          i = _pollInputs(sortedDictValues(self.inputs), t, self.checklevel) 
853          return apply(getattr(self, self.funcspec.spec[1]), [t, x, p+i])

854  
 
855  
 


856 -    def Jacobian(self, t, xdict, pdict=None, asarray=True):


857          """asarray is an unused, dummy argument for compatibility with
 
858          Model.Jacobian""" 
859          if self.haveJacobian(): 
860              # also, ensure xdict doesn't contain elements like array([4.1]) instead of 4
 
861              x = [float(val) for val in sortedDictValues(filteredDict(xdict,
 
862                                                                       self.funcspec.vars))] 
863              if pdict is None: 
864                  pdict = self.pars 
865              p = sortedDictValues(pdict) 
866              i = _pollInputs(sortedDictValues(self.inputs), t, self.checklevel) 
867              return apply(getattr(self, self.funcspec.auxfns["Jacobian"][1]), \
 
868                           [t, x, p+i]) 
869          else: 
870              raise PyDSTool_ExistError("Jacobian not defined")

871  
 
872  
 


873 -    def JacobianP(self, t, xdict, pdict=None, asarray=True):


874          """asarray is an unused, dummy argument for compatibility with
 
875          Model.JacobianP""" 
876          if self.haveJacobian_pars(): 
877              # also, ensure xdict doesn't contain elements like array([4.1]) instead of 4
 
878              x = [float(val) for val in sortedDictValues(filteredDict(xdict,
 
879                                                                       self.funcspec.vars))] 
880              if pdict is None: 
881                  pdict = self.pars 
882              p = sortedDictValues(pdict) 
883              i = _pollInputs(sortedDictValues(self.inputs), t, self.checklevel) 
884              return apply(getattr(self, self.funcspec.auxfns["Jacobian_pars"][1]), \
 
885                          [t, x, p+i]) 
886          else: 
887              raise PyDSTool_ExistError("Jacobian w.r.t. parameters not defined")

888  
 
889  
 


890 -    def AuxVars(self, t, xdict, pdict=None, asarray=True):


891          """asarray is an unused, dummy argument for compatibility with
 
892          Model.AuxVars""" 
893          # also, ensure xdict doesn't contain elements like array([4.1]) instead of 4
 
894          x = [float(val) for val in sortedDictValues(filteredDict(xdict,
 
895                                                                   self.funcspec.vars))] 
896          if pdict is None: 
897              pdict = self.pars 
898          p = sortedDictValues(pdict) 
899          i = _pollInputs(sortedDictValues(self.inputs), t, self.checklevel) 
900          return apply(getattr(self, self.funcspec.auxspec[1]), [t, x, p+i])

901  
 
902  
 


903 -    def __del__(self):


904          ODEsystem.__del__(self)

905  
 
906  
 
907  
 
908  # Register this Generator with the database
 
909  
 
910  symbolMapDict = {} 
911  # in future, provide appropriate mappings for libraries math,
 
912  # random, etc. (for now it's left to FuncSpec)
 
913  theGenSpecHelper.add(Vode_ODEsystem, symbolMapDict, 'python') 
914
```

  


| Home | Trees | Indices | Help | PyDSTool |
| --- | --- | --- | --- | --- |

|  |  |
| --- | --- |
| Generated by Epydoc 3.0.1 on Fri May 4 15:24:16 2012 | http://epydoc.sourceforge.net |
